# Cutaneous Leishmaniasis in Khyber Pakhtunkhwa Province of Pakistan: Clinical Diversity and Species-Level Diagnosis

**DOI:** 10.4269/ajtmh.16-0343

**Published:** 2016-11-02

**Authors:** Nazma Habib Khan, Arfan ul Bari, Rizwan Hashim, Inamullah Khan, Akhtar Muneer, Akram Shah, Sobia Wahid, Vanessa Yardley, Brighid O'Neil, Colin J. Sutherland

**Affiliations:** 1 Department of Immunology and Infection, Faculty of Infectious and Tropical Diseases, London School of Hygiene and Tropical Medicine, London, United Kingdom; 2 Department of Zoology, University of Peshawar, Peshawar, Khyber Pakhtunkhwa, Pakistan; 3 Combined Military Hospital, Peshawar, Khyber Pakhtunkhwa, Pakistan; 4 Khyber Teaching Hospital, Peshawar, Khyber Pakhtunkhwa, Pakistan; 5 Kuwait Teaching Hospital, Peshawar, Khyber Pakhtunkhwa, Pakistan

## Abstract

This study primarily aimed to identify the causative species of cutaneous leishmaniasis (CL) in the Khyber Pakhtunkhwa Province of Pakistan and to distinguish any species-specific variation in clinical manifestation of CL. Diagnostic performance of different techniques for identifying CL was assessed. Isolates of *Leishmania* spp. were detected by in vitro culture, polymerase chain reaction (PCR) on DNA extracted from dried filter papers and microscopic examination of direct lesion smears from patients visiting three major primary care hospitals in Peshawar. A total of 125 CL patients were evaluated. Many acquired the disease from Peshawar and the neighboring tribal area of Khyber Agency. Military personnel acquired CL while deployed in north and south Waziristan. *Leishmania tropica* was identified as the predominant infecting organism in this study (89.2%) followed by *Leishmania major* (6.8%) and, unexpectedly, *Leishmania infantum* (4.1%). These were the first reported cases of CL caused by *L. infantum* in Pakistan. PCR diagnosis targeting kinetoplast DNA was the most sensitive diagnostic method, identifying 86.5% of all samples found positive by any other method. Other methods were as follows: ribosomal DNA PCR (78.4%), internal transcribed spacer 2 region PCR (70.3%), culture (67.1%), and microscopy (60.5%). Clinical examination reported 14 atypical forms of CL. Atypical lesions were not significantly associated with the infecting *Leishmania* species, nor with “dry” or “wet” appearance of lesions. Findings from this study provide a platform for species typing of CL patients in Pakistan, utilizing a combination of in vitro culture and molecular diagnostics. Moreover, the clinical diversity described herein can benefit clinicians in devising differential diagnosis of the disease.

## Introduction

Leishmaniasis, caused by protozoan trypanosomatid parasites of genus *Leishmania*, is essentially a neglected tropical disease. The parasites multiply within host macrophages, afflicting skin (cutaneous), mucous membranes (mucocutaneous), or internal organs (visceral).^1^,^2^ About 350 million people live at risk of leishmaniasis in 98 countries across five continents.^3^,^4^ Based on estimates by Alvar and others (2012),^5^ the annual global incidence of visceral leishmaniasis (VL) is 0.2–0.4 million cases compared with 0.7–1.2 million cases for cutaneous leishmaniasis (CL) and mucocutaneous leishmaniasis combined (MCL).

A region extending from central Asia to Middle East contributes 226,200–416,400 of the global CL cases annually. In Pakistan, around 21,000–35,000 cases of both anthroponotic (ACL) and zoonotic (ZCL) forms of CL are reported.^5^ ACL is apparently sporadic and *Leishmania tropica* has been implicated as its causative agent in the country.^6^–^8^ It is commonly reported from urban centers of Punjab, Baluchistan, Azad Jammu Kashmir (AJK), Khyber Pakhtunkhwa (KP) and the surrounding tribal belt, known as the Federally Administrated Tribal Areas (FATA).^5^,^9^ In KP, the northwest province of Pakistan, leishmaniasis is characterized by intermittent epidemics attributed to *L. tropica*.^6^,^8^,^10^ The ZCL form, attributed to infection by *Leishmania major*, is more common further south, being described from rural and semi-urban areas of Punjab, Baluchistan, and Sindh provinces, where its transmission is possibly maintained through reservoir populations in wild mammals, particularly gerbils such as *Rhombomys opimus*.^11^,^12^

There have been several reports from studies in Pakistan of atypical manifestations of the disease either due to unusual sites of lesions or their unusual morphology. Lesions on atypical sites lead to difficulty in differential diagnosis, for example, lips or genitalia (chancriform), toes and fingers (paronychial), pulp of finger (whitlow), eyelid, scalp, palm/sole (palmoplantar), and others.^13^–^16^ Similarly, many unusual clinical configurations of lesions are described including lupoid, keloidal, psoriasiform, erysipeloid, verrucous, zosteriform, tumorous, eczematoid, and acneform and so on.^16^–^19^ These studies of atypical manifestation in Pakistan have failed to provide molecular confirmation of the infecting *Leishmania* species.

This study aimed to assess the demographic and clinical aspects of CL in KP Province of Pakistan. We also set out to identify the causative species of CL in the province and attempted to distinguish any species-specific clinical variants within KP. Different diagnostic approaches comprising microscopy (including histopathology), parasite culture, and polymerase chain reaction (PCR) were compared to evaluate their sensitivity and specificity. In addition, the diagnostic accuracy of presumptive clinical diagnosis, as performed in the participating hospitals, was examined using the above-mentioned diagnostic means.

## Materials and Methods

### Study design and subjects.

The study was conducted in the dermatology outpatient units at Kuwait Teaching Hospital (KWH), Khyber Teaching Hospital (KTH), and Combined Military Hospital (CMH) in Peshawar, KP. CMH commonly catered for army personnel who acquired CL while deployed in the tribal areas of FATA. The other two civilian hospitals treated patients from local and other adjoining regions in the outskirts of Peshawar. Clinical data and biological samples were collected from 125 CL patients (suspected and confirmed) visiting these major health-care facilities of Peshawar from May 2010 to September 2010.

CL patients were recruited for the study based on standard diagnostic procedures practiced at these hospitals. KWH performed clinical diagnosis only. CMH also practiced clinical diagnosis, often followed by confirmatory microscopy (histopathology or exudate smears). At KTH, clinical diagnosis was considered inadequate and a positive microscopy of lesion smears was required before administering treatment. However, for this study, all suspected (microscopy negative) and confirmed CL patients (microscopy positive) from KTH were included. All participants gave written informed consent to participate in the study. Ethics Committees at University of Peshawar (ref. no. 28/Pharm, May 2010) and the London School of Hygiene and Tropical Medicine (LSHTM) (ref. no. 5677, March 2010) approved the study documentation.

### Sample collection and DNA extraction.

Selected patient lesions (the most recent, in case of multiple lesions) were first photographed and standard clinical descriptions for these lesions were obtained from dermatologists. Lesions were punctured with sterile lancets and collected exudates were dispensed in to a biphasic culture medium containing rabbit blood agar and M199 medium (Sigma-Aldrich, Gillingham, UK) supplemented with 10% heat-inactivated fetal calf serum. Positive cultures with *Leishmania* viable promastigotes observed were expanded and cryopreserved before transfer to LSHTM for genetic analysis. Aliquots of each culture were thawed, spun at 2,000 rpm at 4°C for 10 minutes, the pellet washed with phosphate-buffered saline, and DNA extracted using the DNeasy Blood and Tissue Kit (QIAGEN, North Manchester, UK).

At the same time, coarse porosity filter paper discs (Fisher Scientific, Loughborough, UK) were used to obtain lesion and biopsy impressions from each patient. Filter papers were wrapped individually in airtight resealable bags with silica gel and stored at 4°C until further processing: 2–3 sections of the filter papers were punched using a Harris Uni-Core hole punch (at least 2 mm diameter) and DNA extracted using a resin-based Chelex^®^ method.^20^

### Diagnostic PCR.

Three PCR methods, targeting minicircle kinetoplast DNA (kDNA),^6^ ribosomal DNA (rDNA),^21^ or internal transcribed spacer 2 region (ITS2)^22^ were used for *Leishmania* species identification and discrimination in cultures and on filter paper samples. rDNA provided *Leishmania* genus-level identification, whereas kDNA and ITS2 PCRs aided in species-level diagnosis of the samples (Table 1). *Leishmania* species discrimination from cultured parasites was performed using only kDNA PCR, whereas samples on filter papers were subjected to all three diagnostic PCRs.

### Data analysis.

For analysis, KTH and KWH were treated as a single group of civilian hospitals (KTH + KWH, *N* = 54) distinct from CMH, a military hospital (*N* = 71). Calculation of proportions, Pearson χ^2^ test, and comparison of means by the Wilcoxon–Mann–Whitney test were carried out in STATA v.12.

Sensitivity, specificity, positive predictive value (PPV), negative predictive value (NPV), and McNemar's marginal homogeneity were calculated for each diagnostic method.^23^ Since there is no known gold standard outlined for diagnosis of CL, a “consensus standard” was defined for this study. All samples were considered confirmed positives if they were positive parasitologically (by culture or microscopy) or by at least two PCR methods. Samples were considered confirmed negatives if they were negative by all PCR and parasitological methods or when only one PCR method was positive.^24^–^26^

## Results

A total of 125 patients with skin lesions were diagnosed as cases of CL, and treated according to local guidelines and protocols operating in the three participating hospitals. Our study did not contribute to decisions regarding management of these patients.

**Figure 1.**
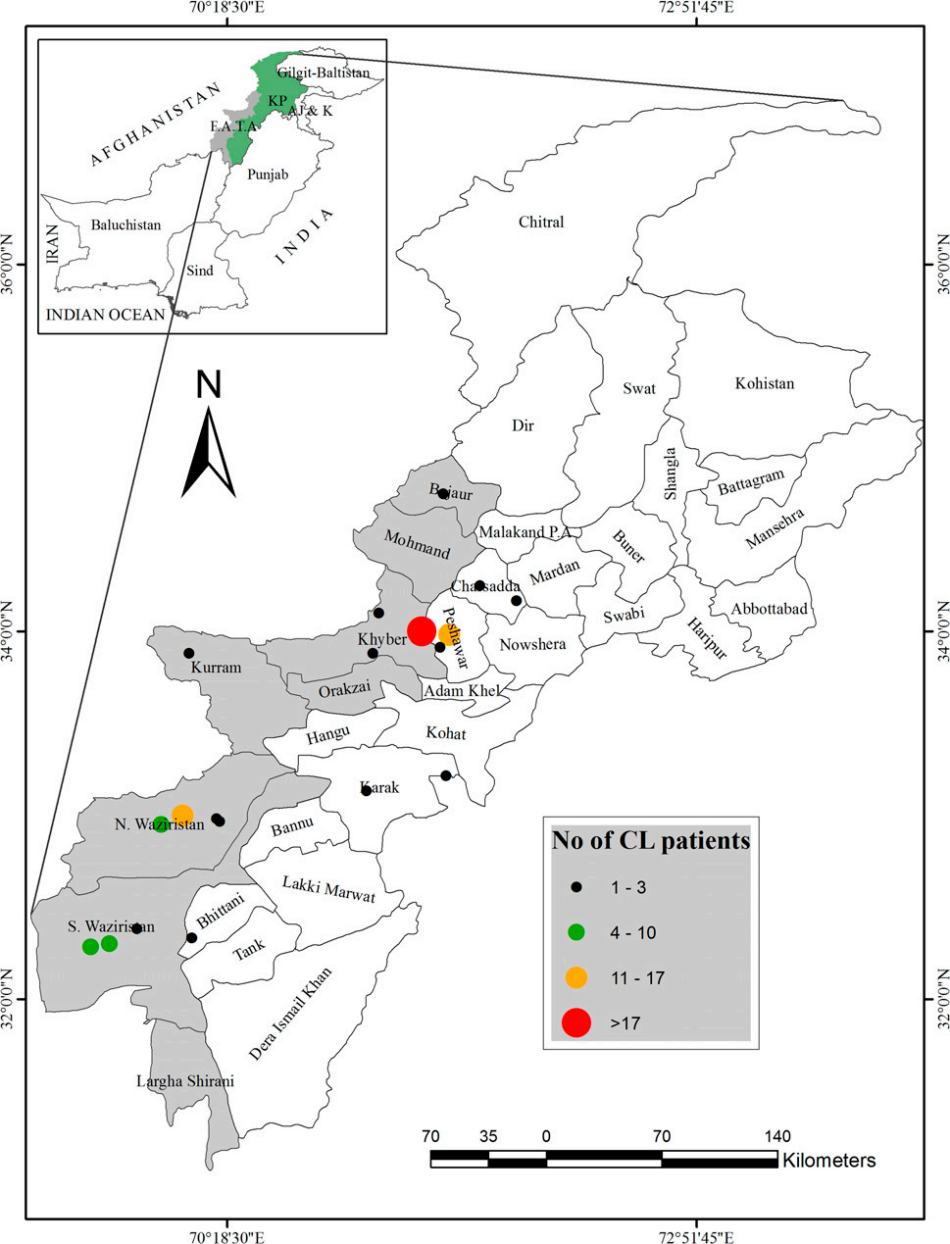
Geographical distribution of cutaneous leishmaniasis (CL) cases reported in the study. Map shows adjoining regions of Khyber Pakhtunkhwa Province. Areas shaded gray are agencies within the Federally Administrated Tribal Areas (FATA). Dot on the map represents the isolation site of one or more strains. Size of the dot is proportional to number of strains represented by it.

Site of disease acquisition was concluded from patient travel history, taking in to account the incubation period and patient information. Overall, 56.1% of patients acquired the disease when traveling/working away from home, both in and outside KP, whereas the rest acquired the disease indigenously. The civilian hospitals (KWH and KTH) mainly received patients from Peshawar and adjoining tribal regions of Khyber Agency (mostly Jamrud, Bara, and Landi Kotal). Military personnel serving in north and south Waziristan (especially Miramshah and Wana) were observed at CMH (Figure 1

).

### Comparative performance of diagnostic methods.

Samples in cultures and on filter papers were successfully acquired from 119 and 111 of 125 patients, respectively. Although 51 of the total 119 lesions were culture positive, only 34 of these could be established and propagated so that they could be further subjected to species-specific PCRs. Microscopy data were provided for 45 of the 71 patients at CMH and all the 16 patients examined at KTH (Table 2). Microscopy was not performed at KWH.

The highest estimated prevalence of *Leishmnania* spp. infection, derived from PCR amplification of clinical samples on filter papers, was 71.2% by rDNA PCR, followed by 62.2% (kDNA), and 46.8% (ITS2). Culture and microscopy methods provided lower estimates of prevalence among the patients tested (42.9% and 42.6%, respectively) (Table 2).

Sensitivity and specificity of each diagnostic PCR assay on filter paper samples was assessed against the consensus standard. kDNA PCR showed the highest sensitivity (86.5%) and specificity (86.5%). This PCR method was also least prone to producing false negatives (NPV = 76.2%). rDNA PCR was the second most sensitive method (78.4%), although suffered from poor specificity (43.2%). ITS2 PCR was the least sensitive (70.3%) but outperformed other methods in specificity (100%). Parasite culture and microscopy provided sensitivity estimates of 67.1% and 60.5%, respectively. Only kDNA and rDNA PCR provided significant statistical equivalence with the consensus standard (McNemar's test; *P* > 0.05) (Table 2).

Clinical examination was a standard means of CL diagnosis at CMH and KWH. About 80.7% (*N* = 88) of the clinically diagnosed CL patients (*N* = 109) were confirmed as parasite positive by at least one other diagnostic method (microscopy, culture, or PCR). On the other hand in KTH, eight of the 16 patients not recommended for treatment (due to negative microscopy) were positive by culture or PCR methods used for the purpose of this study.

### *Leishmania* species in KP, Pakistan.

Of the total of 125 examined patients, 104 were validated *Leishmania* cases by one or more of the diagnostic methods used in this study. Thirty were identified to genus level (positive only by microscopy, culture, or rDNA PCR).

Of the 74 samples identified to species level, 66 were *L. tropica* (89.2%), five were *L. major* (6.8%), and three were *Leishmania infantum*. Three of the *L. major* patients had recently traveled to Baluchistan, whereas the other two had travel histories to Punjab and Sindh. To date *L. infantum* has not previously been reported to cause CL in Pakistan. Two of these cases were army officers who contracted the disease in north Waziristan. The third case was a female of 60 years diagnosed at AJK with no long-term travel history except to seek medical attention (Table 3).

### Disease description and clinical diversity.

**Figure 2.**
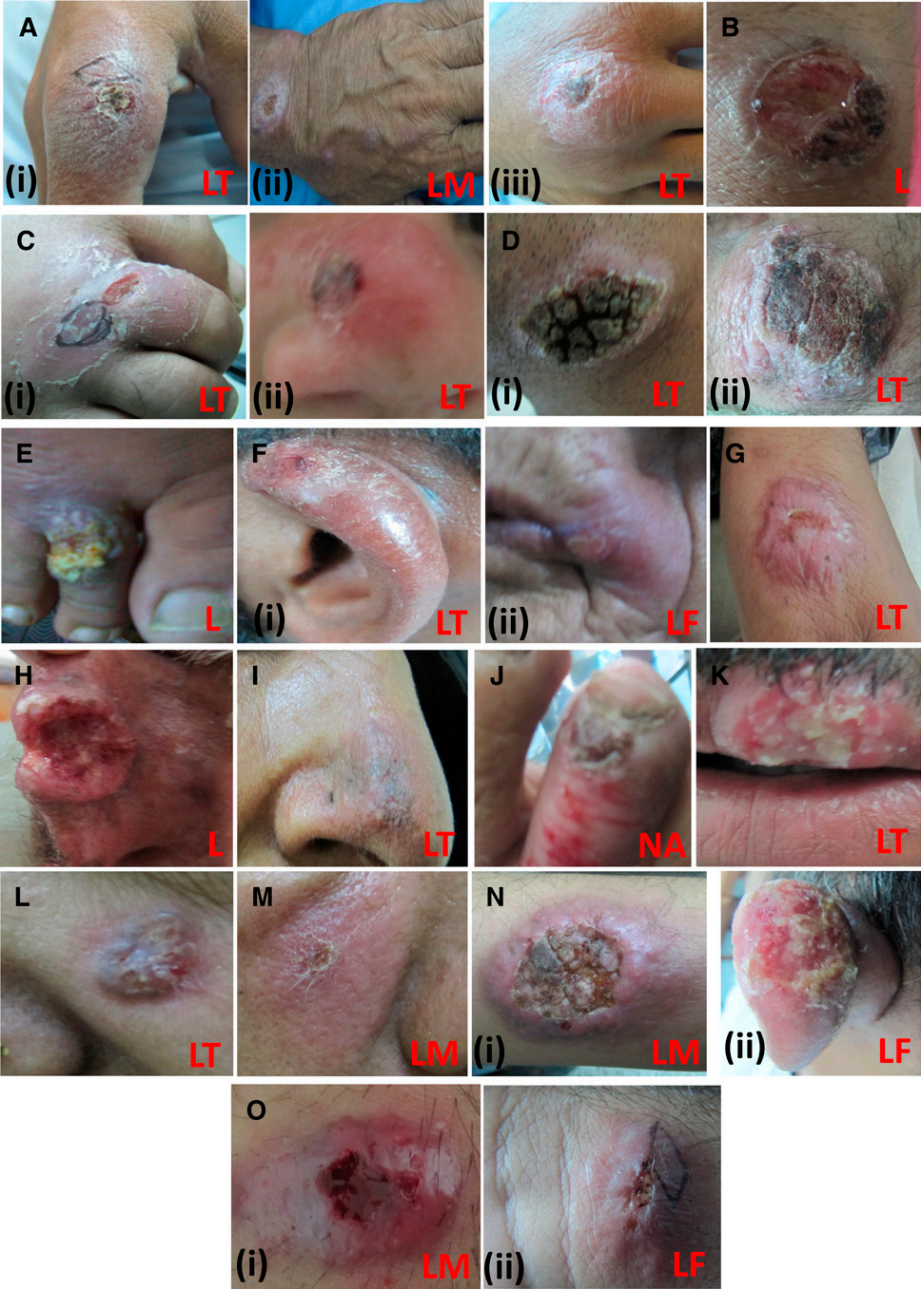
Atypical forms of cutaneous leishmaniasis (CL) lesions reported. (**A**) Psoraisiform, (i and ii) dry type, (iii) mixed type; (**B**) ecthymatous, mixed-type; (**C**) (i and ii) cellulitis like, mixed type. (**D**) (i and ii) Verruciform, dry type; **(E**) mycetomatous, mixed type; (**F**) lupoid, (i) mixed type and (ii) dry type; (**G**) keloidal, dry type; (**H**) squamous cell carcinoma like, wet type; (**I**) discoid lupus erythematosus like, dry type; (**J**) paronychial, dry type; (**K**) chanciform, wet type; (**L**) basal cell carcinoma like, mixed type; (**M**) erysipeloid, dry type; (**N**) typical (i and ii) wet type; and (**O**) typical (i and ii) mixed-type. LT = *Leishmania tropica*; LM = *Leishmania major*; LF = *Leishmania infantum*; L = *Leishmania* spp.; NA = no spp. identified.

Clinical profiles for all the patients in the study (*N* = 125) were assessed for different parameters as seen in Tables 3 and 4. Additional clinical characteristics studied were based on lesion descriptions as provided by the clinician. A total of 99 lesions were represented by photographs of sufficient quality to be adequately described. Briefly, the description included whether the lesion was dry, wet, or mixed types; if ulcerated whether typical or atypical leishmaniasis lesion; if atypical, its description based on its similarity to a certain dermatological condition (Figure 2

).

Duration of disease and size of lesion at presentation did not significantly vary between the two hospital types (Mann-Whitney Wilcoxon test, *P* > 0.05). Presence of singular lesions was significantly higher in the civilian hospitals (Pearson χ^2^, *P* = 0.004). Upper extremities were the most common lesion site (41.6%). Most of the presented lesions were dry type (48.5%) followed by mixed (37.4%) and wet type (14.1%) (Table 4). The findings suggested that infecting species did not affect the number of lesions per patient, nor the dry/wet/mixed presentation (Table 3).

About 26.2% of ulcerated lesions (*N* = 84) presented typical morphology for CL (central crust with raised indurated edges) as described previously.^35^ Among the rest, fourteen atypical forms were described (Figure 2). All the lesions with Cellulitis-like atypical morphology were caused by *L. tropica* (*N* = 6). No conclusions could be drawn for species specificity for other atypical types, as they were present in more than one *Leishmania* species, one or more of their samples were PCR negative, or were identified to genus level only (Supplemental Table 1).

## Discussion

Old World CL is a largely neglected disease. In most studies, the causative agent is either not reported or a particular species is assumed by convention to be present based on previously reported investigations. In this study, a combination of clinical, in vitro, and molecular diagnostic methods show that although three different species of *Leishmania* were found in the study area, the diversity of clinical presentations encountered could not be explained by species differences.

### Demographic characteristics of CL patients.

Overall, the patients seen were predominantly male, and many of them were diagnosed and treated at a military hospital. Civilian hospital patients were also more likely to be males, occurring with twice the frequency of females. Similar sex-wise differences have been previously reported from other primary care hospitals of Peshawar^15^,^27^ and from active studies in neighboring Kabul, Afghanistan.^28^ These observations may indicate that men are at higher odds of acquiring CL, resulting from their occupational exposure and activities such as traveling or the practice of sleeping outside.^29^,^30^ In addition, there may also exist differences in attitudes toward seeking and providing treatment.^31^

Both local hospitals observed high proportions of CL in people from western tribal regions of the province, particularly the Khyber Agency, and also among local Peshawaris. Khyber Agency borders Afghanistan with frequent movement of people to and from Kabul City. These individuals, probably exposed to higher transmission intensity across the border, provide reservoirs for the consequent anthroponotic transmission of the disease when they return to their home regions in Pakistan. The mean age of leishmaniasis patients in both the civilian and military centers (CMH = 28.5, KTH + KWH = 20.8) was in general agreement with other CL studies in the region.^15^,^27^ However, the civilian centers observed a majority of patients under 24 years of age, suggesting long-term development of immunity in a region (Khyber Agency) with established endemicity for the disease,^32^ or perhaps different treatment-seeking behavior in older age groups.

### Proficiency of different diagnostic methods in KP.

Comparing the diagnostic performance of different methods in this study showed that both the sensitivity of microscopy and the isolation rate of culture were satisfactory at 66% and 67%, respectively (Table 2). Previous studies in KP observed lower sensitivity of these techniques (36–43%) compared with parasite culture.^8^ However, countries from Latin America and central Asia witnessed a better performance of microscopy against PCR (78–90%).^33^–^35^ A major issue with culture-based diagnosis for CL is the high risk of contamination,^36^ as was observed in this study (9.2% of cultured isolates). Several factors may account for such variations in sensitivities including low parasite burden (e.g., chronic CL) and variation in number and distribution of the parasites in samples.^37^,^38^

Filter papers have been used as source of clinical material in Afro-Eurasian CL, American CL, MCL, and extensively in VL studies.^24^,^39^–^41^ Using filter paper as clinical samples, kDNA-targeted PCR was the most sensitive of all the methods used, as corroborated by others.^24^ kDNA assays are demonstrated to be more sensitive than PCR of rDNA targets due to higher copy number (10, 000 minicircles per parasite as compared with 40–200 copies of the rDNA genes).^6^,^24^,^42^ Poor performance of ITS2 observed herein might be attributed to PCR inhibition since nested amplification from rDNA PCR was used. Studies have, however, demonstrated low sensitivity of ITS2 PCR on clinical samples (21.2%).^43^,^44^ Sequence variation at primer sites may play a role here. Lack of PCR positivity in some parasitologically positive specimens might have been due to heterogeneous distribution of the parasite across the filter paper. This condition would be exacerbated in cases of low parasitemia.

Presumptive clinical diagnosis, as performed in the participating hospitals, was shown to be effective in this endemic setting.^8^ Thus, although several atypical forms of the disease were prevalent, experienced local clinicians provided diagnoses of high accuracy, a reassuring finding since microscopy has the limitations of decreased sensitivity and dependence on trained technical staff.^3^

Our combined diagnostic approach identified three parasite species as causative agents of leishmaniasis in our study hospitals: *L. tropica* (89.2%), *L. major* (6.8%), and *L. infantum* (4.1%). *Leishmania tropica* has been previously identified as the causative agent of ACL in KP, neighboring AJK, Quetta, Baluchistan, and south Punjab.^7^,^8^,^45^,^46^ All *L. tropica* samples were isolated from patients from KP province, substantiating the endemicity of the species in this region.^6^–^8^ All five *L. major* cases in this study were probably imported into the region, since the patients had travel histories to regions from Baluchistan, Punjab, and Sindh. All these provinces have endemic gerbil populations, and are suspected or known to have *L. major* transmission.^46^–^48^ However, this does not overlook possible ZCL transmission of *L. major* in KP, since its incriminated vector *Phlebotomus papatasi* exists here although not as widespread as in Baluchistan.^49^ This study reported the first cutaneous cases of *L. infantum* in Pakistan. Although one patient contracted the disease in AJK, which is a focus of VL by *L. infantum*,^50^–^52^ the other two cases reported here were from KP. There are no reports of *L. infantum* causing CL circulating in KP. As the patients in this study were not examined for symptoms of VL, we cannot rule the possibility that VL does occur in our study area.

### Clinical features of CL in KP.

The presence of singular lesions was significantly associated with the region of disease origin and type of hospital. Housing conditions and vocational behavior of the patients may account for the variation in number of lesions, since the military personnel visiting CMH live in tents and barracks and had duty hours making them more susceptible to multiple bites. A probable explanation can also lie in host immunity toward the disease as it has been observed that nonindigenous people are prone to having acute, severe, and more lesions than seen in indigenous cases.^53^ Mean lesion duration and lesion size at presentation were 3.5 months and 2.02 cm, respectively. The reported values were comparable to those presented by others (6–10 months; 1.2–1.7 cm).^35^,^54^,^55^ Overall, the most commonly afflicted sites effected by CL were exposed upper extremities and face of patients since they are the more consistently exposed parts.^9^,^28^,^35^,^55^

The majority of *L. tropica* lesions observed here were dry or mixed type lesions. *Leishmania tropica* has been previously associated with production of dry type lesions, whereas wet/moist lesions are reported to be initiated by *L. major* infections.^56^,^57^ Studies in *L. tropica*–endemic regions of the country extensively report dry type lesions (in KP 57.6% by Bari and others^54^; in Multan, Punjab, 100% by Mujtaba and Khalid^58^; Larkana, Sindh, 97.6% by Bhutto and others^12^). Clinically dry and wet types of lesions were observed in all the three species detected in this study and we thus found no evidence of species-specific clinical manifestations. Similar conclusions were also drawn by Myint and others^46^ in Baluchistan. We also failed to find any evidence that atypical lesion morphology could be associated with a specific *Leishmania* species.^16^,^17^,^19^

It is important to consider that the clinical outcome of CL does not only depend on the *Leishmania* species responsible. Rare clinical manifestations may be affected by host immune response, nutritional status of the host, hormonal factors, or differences in size and site of inoculation. Unusual morphology related to atypical site might also be because of variation in the skin barrier (e.g., fragility of facial skin) or the topography of the site of the sand fly bite (e.g., Fissure leishmaniasis when the lesion is in the center point of the lip, on the face^59^).

## Conclusion

Our study provides a platform for future species typing of CL patients in KP and other provinces, utilizing a combination of in vitro culture and molecular diagnostics. Identifying potential hosts and vectors for *L. infantum* in the region now must be addressed.

## Supplementary Material

Supplemental Table.

## Figures and Tables

**Table 1:** Primers and reaction conditions used in diagnostic PCRs

PCR	Forward (F) primer/reverse (R) primer (5′–3′)	PCR mixture	Cycling conditions
rDNA^21^	Nest1 F: GCTGTAGGTGAACCTGCAGCAGCTGGATCATT	50 μL reaction; 5 μL DNA from filter papers (or 5 μL N1), 25 μL QIAGEN HotStar Taq Master Mix (QIAGEN), 0.3 μM primers	95°C, 15 minutes
	Nest1 R: GCGGGTAGTCCTGCCAAACACTCAGGTCTG		35–40 cycles
	Nest2 F: GCAGCTGGATCATTTTCC		94°C, 0.5 minutes
	Nest2 R: AACACTCAGGTCTGTAAAC		58°C, 0.5 minutes
			72°C, 1.5 minutes
			60°C, 1 minutes
			75°C, 10 minutes
ITS2^22^	F: GGGAGAAGCTCTATTGTG	25 μL reaction; 1 μL N2 product from rDNA PCR, KCl buffer (15 mM MgCl2, Bioline, London, UK), 2 mM deoxynucleotide triphosphates, 0.4 μM primers, 1U Taq polymerase	94°C, 2 minutes
	R: ACACTCAGGTCTGTAAAC		40 cycles
			94°C, 20 seconds
			53°C, 30 seconds
			72°C, 1 minutes
			72°C, 10 minutes
kDNA^6^	Nest1 F: C/GA/GTA/GCAGAAAC/TCCCGTTCA	50 μL reaction; 5 μL DNA from filter papers (OR 5 μL N1), 25 μL QIAGEN HotStar Taq Master Mix, 0.3 μM primers	95°C, 15 minutes
	Nest1 R: ATTTTTCG/CGA/TTTT/CGCAGAACG		30 cycles at
	Nest2 F: ACTGGGGGTTGGTGTAAAATAG		94°C, 1 minutes
	Nest2 R: TCGCAGAACGCCCCT		55°C, 1 minutes
			72°C, 1.5 minutes
			60°C, 1 minutes
			75°C,10 minutes

kinetoplast DNA = kDNA; PCR = polymerase chain reaction.

**Table 2:** Comparative performance of diagnostic methods used to identify *Leishmania* infections among 125 patients

Diagnostic method	No. of positives (% positivity)	Sensitivity (%) (95% CI)	Specificity (%) (95% CI)	PPV (%)	NPV (%)	*P* value*
kDNA PCR(*N* = 111)	69 (62.2)	86.5 (76.5–93.3)	86.5 (71.2–95.5)	92.8	76.2	0.197
ITS2 PCR(*N* = 111)	52 (46.8)	70.3 (58.5–80.3)	100	100	62.7	< 0.01
rDNA PCR(*N* = 111)	79 (71.2)	78.4 (67.3–87.1)	43.2 (27.1–60.5)	73.4	50	0.411
Microscopy (*N* = 61)†	26 (42.6)	60.5 (44.4–75)	100	100	51.4	< 0.01
Culture (*N* = 119)†‡	51 (42.9)	67.1 (55.4–77.5)	100	100	63.2	< 0.01

CI = confidence interval; ITS2 = internal transcribed spacer 2 region; kinetoplast DNA = kDNA; NPV= negative predictive value; PCR = polymerase chain reaction; PPV = positive predictive value; rDNA = ribosomal DNA.

* For McNemar's test of marginal homogeneity.

† Both the methods contributed to formulating the “consensus” standard as no gold standard method exists.

‡ For culture, sensitivity figure is equivalent to isolation rate.

**Table 3:** Types and number of lesions in relation to *Leishmania* species identified in the region

Dry/wet* (%)	*Leishmania tropica* (*N* = 59)	*Leishmania major* (*N* = 4)	*Leishmania infantum* (*N* = 3)	*P* value†
Dry	24 (40.7)	2 (50.0)	1 (33.3)	0.171
Mixed	28 (47.5)	0	1 (33.3)	
Wet	7 (11.9)	2 (50.0)	1 (33.3)	
No. of lesions (%)	*L. tropica* (*N* = 66)	*L. major* (*N* = 5)	*L. infantum* (*N* = 3)	
Single	38 (57.6)	2 (40.0)	3 (100)	0.241
Multiple	28 (42.4)	3 (60.0)	0	

* Includes only those samples for which clinical description was available.

† Calculated for Pearson χ^2^ test.

**Table 4:** Patient information and clinical data from lesions

	Study center			*P* value*
	All (*N* = 125)	CMH (*N* = 71)	KTH + KWH (*N* = 16 + 38 = 56)	
Median age of patients (interquartile range)	24 (17–32)	26 (22–32)	16 (7–30)	–
Sex of patients				
Male (%)	84.8	97.2	68.5	–
Female (%)	15.2	2.8	31.5	–
No. of lesions per patient				
Mean	2.2 (95% CI 1.4–2.9)	2.6 (95% CI 1.2–3.9)	1.6 (95% CI 1.3–2.0)	–
Range	1–47	1–7	1–47	–
Singular lesion (%)	59.7	48.6	74.1	0.004
Multiple lesion (%)	40.3	51.4	25.9	
Duration of disease at presentation (months)†				
Mean	3.5 (95% CI 3.1–4.0)	3.4 (95% CI 3.0–3.9)	3.7 (95% CI 2.8–4.6)	0.362
Range	0.36–12	0.75–10	0.36–12	
Size of lesion at presentation (cm)‡				
Mean (*N* = 108)	2.0 (95% CI 1.8–2.2)	2.1 (95% CI 1.8–2.4)	2.0 (95% CI 1.7–2.3)	0.392
Range	0.5–6	0.5–6	0.5–6	
Site (%)§				
Upper extremity	41.6	47.9	33.3	0.102
Lower extremity	24.8	21.1	29.6	0.276
Lesions on both extremities	10.4	15.5	3.7	0.032
Lesions on and above neck	29.6	23.9	37.4	0.112

CI = confidence interval; CMH = Combined Military Hospital; KTH = Kuwait Teaching Hospital; KWH = Khyber Teaching Hospital.

* Calculated for Pearson χ^2^ test except for lesion duration and lesion size where the *P* values are for Wilcoxon–Mann–Whitney test.

† Earliest appearing lesion was considered in case of multiple lesions.

‡ Size of lesion that was selected for sampling. The analysis excludes lesions that could not be measured due to absence of clearly defined lesion boundaries (*N* = 17).

§ Percentages will not add up to 100% because of patients with multiple lesions at multiple sites (e.g., on upper extremities and face).
