# Supplementary material for: Cutaneous Leishmaniasis in Khyber Pakhtunkhwa Province of Pakistan: Clinical Diversity and Species-Level Diagnosis
Source: Am J Trop Med Hyg. 2016 Nov 2;95(5):1106–14. doi: 10.4269/ajtmh.16-0343 (PMC5094225; doi:10.4269/ajtmh.16-0343)
Supplement: Supplementary file 1 [file SD5.pdf]

Supplemental Table 1

Leishmania species identified among ulcerated lesions (with typical and atypical presentations)

	Leishmania tropica	*Leishmania major*	*Leishmania infantum*	*Leishmania* genus	Negative
Typical ulcerated lesion (*N* = 22)	12	2	2	5	1
Psoriasiform (*N* = 37)	19	1	0	11	6
Cellulitis like (*N* = 6)	6	0	0	0	0
Verruciform (wart-like) (*N* = 5)	3	0	0	2	0
Echthymatous (*N* = 3)	1	0	0	1	1
Lupoid (*N* = 2)	1	0	1	0	0
Tumorous (squamous-cell carcinoma like) (*N* = 2)	0	0	0	1	1
Keloidal (*N* = 1)	1	0	0	0	0
Discoid lupus erythematosus like (*N* = 1)	1	0	0	0	0
Paronychial (*N* = 1)	0	0	0	0	1
Erysipeloid (*N* = 1)	0	1	0	0	0
Mycetomatous (*N* = 1	1	0	0	0	0
Basal cell carcinoma like (*N* = 1)	1	0	0	0	0
Chancriform (*N* = 1)	1	0	0	0	0
